# Supplementary material for: Factors for Predicting Noninvasive Ventilation Failure in Elderly Patients with Respiratory Failure
Source: J Clin Med. 2020 Jul 4;9(7):2116. doi: 10.3390/jcm9072116 (PMC7408979; doi:10.3390/jcm9072116)
Supplement: Supplementary file 1 [file jcm-09-02116-s001.pdf]

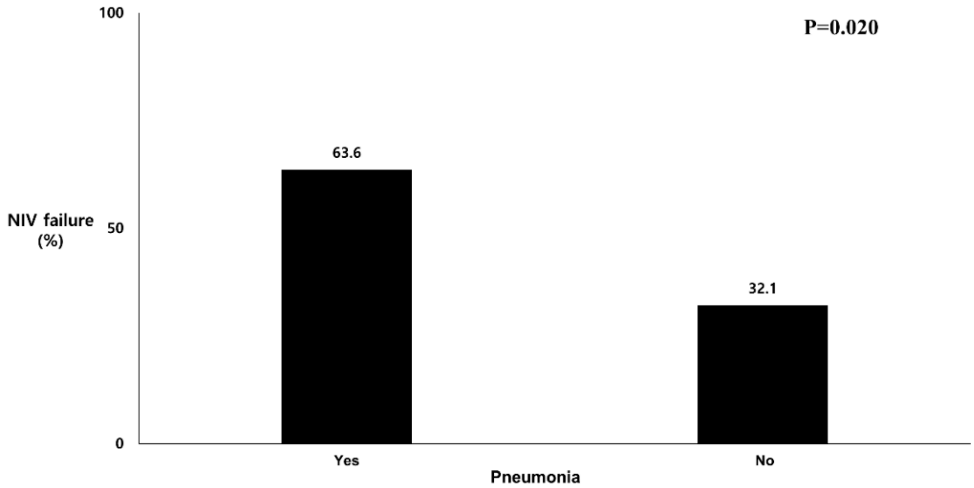

Figure S1. Association between pneumonia at admission and NIV failure. Patients with pneumonia at admission were significantly more likely to experience NIV failure compared to those without pneumonia.

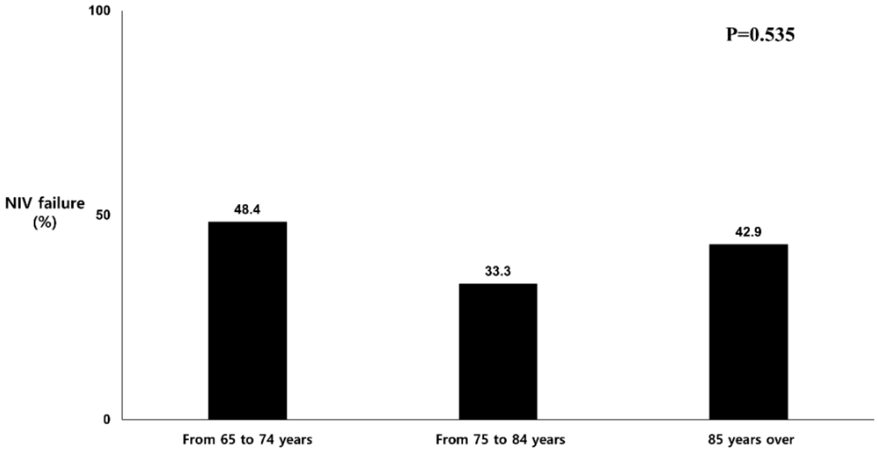

Figure S2. The association between age subgroup and noninvasive ventilation failure. Age subgroup (65–74 years, 75–84 years, and 85 years over) was not associated with NIV failure (p = 0.535).

Table S1. Changes in physiological parameters after NIV commencement in patients with hypercapnic respiratory failure alone.

**Variables**	**NIV application (n = 54)**		**p-Value**
	Before	After	
NIV Success (n = 35)			
Systolic blood pressure(mmHg)	123 (105–141)	117 (105–129)	0.144
Heart rate (beats/min)	91 (82–105)	89 (72–98)	0.019
Respiratory rate (breaths/min)	23 (20–28)	22 (19–26)	0.534
Arterial pH	7.33 (7.28–7.38)	7.37 (7.30–7.43)	<0.001
PaO_2/FiO_2 ratio	227 (140–287)	235 (179–271)	0.451
$PaCO_2$ (mmHg)	71 (60–83)	64 (53–76)	<0.001
NIV failure (n = 19)			
Systolic blood pressure(mmHg)	132 (123–151)	132 (123–146)	0.721
Heart rate (beats/min)	107 (90–112)	96 (85–114)	0.131
Respiratory rate (breaths/min)	26 (23–30)	22 (19–28)	0.161
Arterial pH	7.31 (7.24–7.4)	7.36 (7.32–7.42)	0.008

PaO_2/FiO_2 ratio	225 (152–273)	229 (191–280)	0.596
$PaCO_2$ (mmHg)	63 (50–79)	50 (44–67)	<0.001

Abbreviations: NIV, non-invasive ventilation; PaO_2, arterial partial pressure of oxygen; FiO_2, fraction of inspired oxygen; $PaCO_2$, arterial partial pressure of carbon dioxide. Data are presented as median (25th percentile–75th percentile). The Wilcoxon signed-rank test was used to determine NIV effect for physiologic parameters and laboratory parameters after NIV application.
